# Dynamics of task-induced modulation of spontaneous brain activity and functional connectivity in the triple resting-state networks assessed using the visual oddball paradigm

**DOI:** 10.1371/journal.pone.0246709

**Published:** 2021-11-04

**Authors:** Hasan Sbaihat, Ravichandran Rajkumar, Shukti Ramkiran, Abed Al-Nasser Assi, N. Jon Shah, Tanja Veselinović, Irene Neuner

**Affiliations:** 1 Institute of Neuroscience and Medicine, INM-4, Forschungszentrum Jülich GmbH, Jülich, Germany; 2 Department of Medical Imaging, Arab-American University Palestine, AAUP, Jenin, Palestine; 3 Department of Psychiatry, Psychotherapy and Psychosomatics, RWTH Aachen University, Aachen, Germany; 4 JARA–BRAIN–Translational Medicine, Aachen, Germany; 5 Department of Neurology, RWTH Aachen University, Aachen, Germany; 6 Institute of Neuroscience and Medicine, INM-11, Forschungszentrum Jülich GmbH, Jülich, Germany; Temple University, UNITED STATES

## Abstract

The default mode network (DMN), the salience network (SN), and the central executive network (CEN) are considered as the core resting-state brain networks (RSN) due to their involvement in a wide range of cognitive tasks. Despite the large body of knowledge related to their regional spontaneous activity (RSA) and functional connectivity (FC) of these networks, less is known about the dynamics of the task-associated modulation on these parameters and the task-induced interaction between these three networks. We have investigated the effects of the visual-oddball paradigm on three fMRI measures (amplitude of low-frequency fluctuations for RSA, regional homogeneity for local FC, and degree centrality for global FC) in these three core RSN. A rest-task-rest paradigm was used and the RSNs were identified using independent component analysis (ICA) on the resting-state data. The observed patterns of change differed noticeably between the networks and were tightly associated with the task-related brain activity and the distinct involvement of the networks in the performance of the single subtasks. Furthermore, the inter-network analysis showed an increased synchronization of CEN with the DMN and the SN immediately after the task, but not between the DMN and SN. Higher pre-task inter-network synchronization between the DMN and the CEN was associated with shorter reaction times and thus better performance. Our results provide some additional insights into the dynamics within and between the triple RSN. Further investigations are required in order to understand better their functional importance and interplay.

## Introduction

Examination of regional spontaneous brain activity (RSA) and functional connectivity (FC) during resting-state (RS) conditions appears to be a promising approach for understanding brain organization at the systems level [1]. Within the several stable RS networks identified up to now, three networks stand out for their importance and synchronized interplay: the default mode network (DMN), the salience network (SN), and the central executive network (CEN). These networks are often jointly referred to as the triple network model [2] and are considered to be the core neurocognitive networks due to their involvement in a wide range of cognitive tasks [1,3,4].

Specifically, the DMN is known to be a task-negative network associated with self-referential thoughts and mind-wandering [5]. It shows decreased activation during tasks in which self-referential and stimulus-independent intellectual activity is not involved [6,7]. Even more, numerous studies have demonstrated that midline DMN regions are among the most efficiently wired brain areas, serving as global hubs that bridge different functional systems across the brain [8,9]. Increased DMN connectivity with regions of other brain networks has been shown to facilitate performance during goal-directed tasks [10]. Thus, DMN is not engaged only under resting-state conditions but also under task performance and post-task processes as well [10–12].

The CEN is a task-positive network, engaged in higher-order cognitive and attention control as well as in working memory, decision making and goal-directed behavior [13–15]. Conversely, the SN is involved in detecting, filtering and integrating relevant internal (e.g., autonomic input) and external (e.g., emotional information) salient stimuli in order to guide behavior [1,16]. Furthermore, it displays a crucial role in the functional and dynamic switching between the DMN and CEN (i.e., between task-based and task-free states) [17,18].

Dynamic interactions between the three networks of the triple network model influence cognition and emotion, affecting performance and impulsivity [19–21]. Moreover, an altered interaction between these networks has been shown in patients with major depressive disorder [22], post-traumatic stress disorder [23], obsessive-compulsive disorder [24], and schizophrenia [25,26]. Altogether, an increasing body of evidence suggests that aberrant function of the triple networks underlies the psychopathology of all major psychiatric disorders [27] and disturbed functional interactions among them may be considered a potential neurophysiological biomarker for different psychopathological phenomena across several neuropsychiatric disorders [28]. It is therefore particularly important to understand the physiological fluctuations in the activity and interactions of these networks in order to be able to differentiate them from pathological conditions.

Continuous fluctuations of the main properties of the networks (as RSA and FC) have been shown during rest and during task-associated activities [29,30]. Much less is known about the extent to which these properties can be influenced by a specific task and to what extent a task-associated activity affects the interaction between the networks.

A simple method to investigate the effects of task-related activation on the RSA is the rest-task-rest paradigm (RTR) [5,31]. To date, a task-induced modulation of the RSA has been observed following cognitive tasks involving working memory, emotion, visual perception, and motor training. However, previous studies have mainly focused on whole-brain [31–35] or on specific brain structures known to be involved in the tasks [36,37]. None of the mentioned studies has specifically addressed the impact of a task on the triple network. Moreover, previous investigations have overall reported changes in static connectivity in different time periods (before and after the task), but the dynamic of the changes during the task performance as well as changes in the relationship between the different networks (particularly in the triple network, which is the focus of this study) remain poorly understood. Thus, in this study, we have specifically examined the dynamic of the task-induced changes in RSA and FC within the triple network of the RS (DMN, SN and CEN) and the task-induced effects on the interactions between them.

Concretely, this study aims to assess the dynamics of the influence exerted by a well-established task—the visual oddball paradigm (VOP) [38]—on the triple RS networks using a RTR design. The VOP was chosen as it elicits the blood oxygen level dependent (BOLD) response in a large set of distributed networks [39–43]. In particular, the task performance is associated with activation in brain regions linked to the three networks (the SN [44], the dorsolateral prefrontal cortex (CEN) [45,46], and the cingulate and prefrontal cortex (DMN) [47]).

For the identification of the triple network regions, we applied a group independent component analysis (ICA) to the RS data. Several different measures of FC can be calculated from fMRI, each reflecting a different property of the brain networks. For this approach, we chose two such measures, the regional homogeneity (ReHo) [48] and the degree centrality (DC) [49], as these are suitable for investigating the voxel level local and global FC, respectively. Furthermore, the amplitude of low-frequency fluctuations (ALFF) [50], is suitable for depicting the RSA. Combining these measures enables the complementary characterization of changes in activation and communication of specific networks or regions.

We hypothesized that the task-based activity would distinctly affect the RS RSA as well as the local and the global connectivity in the triple network. Due to the central role of the SN during the occurrence of salient stimuli or during the performance of a cognitive task, we also expected internetwork functional connectivity to increase between the SN and the other two networks of the triple network model (DMN, CEN).

## Materials and methods

### Subjects

21 right-handed healthy subjects (17 males and 4 females) were included in this study (age range between 19 to 40 years; mean: 29 ± 5.6 years). The possible influence of gender was not further investigated in this study due to the large imbalance between the number of men and women and the relatively small sample size. Moreover, a mutual gender effect was not the focus in the study design presented. All subjects were healthy and without a history of neurological or psychiatric disorders. The study was approved by the Ethics Committee of the Medical Faculty of the RWTH Aachen University, Germany. Written informed consent was obtained from all subjects following the recommendations of the Declaration of Helsinki.

### Experimental design

**Fig 1:**
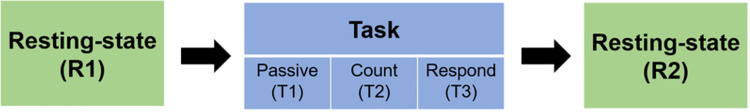
Experimental design of the rest-task-rest paradigm (RTR) which includes two resting-state conditions (pre- and post-task resting-state, R1 and R2) and the task condition composed of three subtasks (passive (T1), count (T2) and response (T3)) of the visual oddball paradigm (VOP).

To investigate the effects of task-induced brain activity on the post-task resting-state, the experiment followed a rest-task-rest (RTR) design consisting of five different conditions: first RS (R1), active state (the visual oddball paradigm (VOP) comprised of three subtasks: passive (T1), count (T2), and respond (T3)) and the second, post-task RS (R2) (Fig 1).

During the two RS conditions, the subjects were instructed to close their eyes and not to focus on any specific thoughts. All fMRI data were acquired in a single scanning session and instructions were given to the subjects in-between each condition via a microphone. Both RS conditions lasted 6 minutes.

The VOP comprised of three subtasks: passive (T1), count (T2), and respond (T3). Each subtask lasted 10 minutes and 8 seconds. In the task, blue circles were used as targets while yellow circles represented frequent stimuli. Each subtask included 200 trials (160 frequent and 40 target stimuli). The single stimulus was 30 cm in diameter shown on a black background for 500 milliseconds with a variable interstimulus interval (ISI) of 500–10,000 milliseconds. The stimulus generator board (ViSaGe MKII, Cambridge Research System Ltd.) was used to generate the stimuli and a thin-film transistor display was used to view the stimuli. The thin-film transistor display was installed behind the scanner and was viewed using a mirror placed on the head coil of the magnetic resonance (MR) scanner. The order of the stimuli during the single subtasks were generated using optseq (http://surfer.nmr.mgh.harvard.edu/optseq).

During T1 the subjects were asked to simply keep the stimuli under observation. During T2 they were asked to count the target stimuli (blue circles) and report the number at the end of the run. During T3 they were instructed to press a button (Lumitouch, Photon Control Inc., Burnaby, BC, Canada) using their right index finger as soon as they recognize the target stimuli. Instructions were given before each imaging sequence and the measurement started immediately following the instructions, without any time delay and without a break between the individual sections.

The passive condition was performed in the beginning for all subjects to exclude any bias caused by the previous knowledge about the blue circles as the targets. The order of the count and respond conditions was counterbalanced across subjects. In general, T1 is suitable for capturing effects of pure sensory perception of the stimuli, while T2 includes an additional cognitive process (counting the target stimuli) and T3 also records effects of the motor response.

A part of this data set (N = 16), which mainly focused on the analysis of the effects of different response modalities on the fMRI BOLD activation during the visual oddball paradigm, has been published previously [51]. Further details on the paradigm are also available in this publication.

### MR data acquisition

MR data were acquired using a 3T scanner (TIM-Trio, Siemens Healthineers, Erlangen, Germany). Sponge pads were used to reduce motion artefacts by limiting the subject’s head movement. The fMRI data were acquired using an echo planner imaging (EPI) sequence. The number of volumes were 304 for each task and 180 for each RS condition (repetition time (TR) = 2000 ms, echo time (TE) = 30 ms, flip angle (FA) = 79°, field of view (FOV) = 200 × 200 mm, 64 × 64 matrix, slice thickness = 3 mm, number of slices = 33, and voxel size 3.1 × 3.1 × 4.2 mm).

Structural images were acquired using a magnetization prepared rapid gradient echo (MP-RAGE) sequence (TR = 2250 ms, TE = 3.03 ms, FA = 9°, FOV = 256 × 265 mm, 64 × 64 matrix, 176 slices, voxel size 1 × 1 × 1 mm).

### fMRI data analysis

The preprocessing of the RS and task data was performed in three separate procedures–firstly the triple network identification was performed using ICA, secondly the task data was processed using general linear modelling (GLM) and finally fMRI parameters (ReHo, DC and ALFF) were calculated for all the five conditions. Detailed explanation on each of these procedures is given below.

### Triple network identification

The multivariate exploratory linear decomposition into independent components (MELODIC) tool from the FSL software package was used to identify the triple networks (DMN, CEN, and SN) using pre-task RS fMRI data. Subject level RS-fMRI data were pre-processed as follows: the first eight fMRI volume images were removed, followed by slice timing correction, brain extraction (BET) [52], motion correction (MCFLIRT) [53], spatial smoothing FWHM = 5 mm, and high-pass temporal filtering 125s. The functional MRI images were co-registered linearly to high-resolution structural images and nonlinearly to MNI standard space using FLIRT [54]. Group ICA analysis was used to decompose the pre-task RS data into 20 components.

To identify the triple networks, a cross-correlation was performed between the functional brain networks atlas [55] and each of the ICA components. The cross-correlation was performed using the FSLUTILS (https://fsl.fmrib.ox.ac.uk/fsl/fslwiki/Fslutils) tool implemented in the FSL software package. ICA components that showed maximum correlation with each of the three networks in the functional brain networks atlas were chosen. The identified brain networks were binarized and used in the subsequent analysis as masks. The binarized masks were corrected for grey matter (GM) by including the voxels which showed more than 50% probability of being GM. The GM correction was performed using a tissue segmented MNI152 (2 × 2 × 2 mm) template.

#### Task data

The analysis of the task-related brain activation was performed using FSL software package (FMRIB’s Software Library, www.fmrib.ox.ac.uk/fsl). The pre-processing included slice timing correction, brain extraction (using BET) [52], motion correction (MCFLIRT) [53], spatial smoothing using a Gaussian kernel of full width at half maximum (FWHM) of 5 mm, and high pass temporal filtering (100s). A time-series of BOLD signal based on the general linear model for each individual data set was performed using FILM with local autocorrelation correction [56]. The functional images were registered to the high-resolution structural images and subsequently to the Montreal Neurological Institute (MNI) standard space using the FLIRT tool [54]. The first-level analysis was performed with two explanatory variables (EV). The EVs were convolved with a double-gamma hemodynamic response function (HRF). Four contrasts were then created: target stimuli, frequent stimuli, target > frequent, frequent > target.

Group-level mixed-effects analysis was performed for the passive, count and respond sub-tasks to create a mean for each first level contrast using FLAME with spatial normalization to MNI space. Cluster correction for multiple comparison was performed using FEAT with a cluster significance threshold of Z > 2.3, p = 0.01 [57]. A tripled two-group difference (“tripled t-test”) was performed to evaluate the additional activation added to the passive condition by the count and respond conditions. The activation pattern regions were defined using Harvard-Oxford Cortical Structural Atlas in FSL software (FMRIB, Oxford, UK).

The reaction time (RT) was calculated as the co-registered time delay between the presentation of the stimulus and the time at which the subject responded to the stimuli by pressing the specified button on the Lumitouch.

### fMRI measures calculation

The fMRI measures were computed for both the tasks and RS-fMRI using data processing and analysis for brain imaging (DPABI) [58], and SPM12 (http://www.fil.ion.ucl.ac.uk/spm/) toolboxes built on MATLAB software package version 2017b (The Math Works, Inc., Natick, MA, USA). Pre-processing was performed using the data processing assistant for the RS-fMRI (DPARSF) [59] advanced edition as follows: first eight fMRI volume images of each condition in each subject’s dataset were removed, followed by slice timing correction, realignment, nuisance covariates regression (NCR) and temporal filtering between 0.01 and 0.08 Hz. The covariates for NCR included head motion parameter, whole-brain white matter (WM) and cerebrospinal fluid (CSF) mean signals, and the constant, linear and quadratic trends in the BOLD signals. To ensure the quality of the data and minimize the movement artefacts, the subjects head motion parameters was estimated and corrected for fMRI data using Friston 24-parameter model [60]. The model showed for all subjects a head motion ≤ 2 mm in translation or ≤ 2° in rotation in any direction. The fMRI measures were calculated for each subject separately in individual brain imaging space. The DC was computed by applying a Pearson correlation coefficient between the time series of a given voxel and all other voxels in the whole brain by thresholding each correlation at (r > 0.25, p ≤ 0.001) [61]. ReHo was calculated by estimating the synchronization or similarity between the time series of a given voxel and 26 nearby neighbor voxels [48] using Kendall’s coefficient of concordance (KCC) [62]. The ALFF was calculated within the low-frequency range (0.01–0.1 Hz) [63]. The fMRI measures were normalized using a Z-value standardization procedure by subtracting the mean from each voxel and then dividing the value by the standard deviation of the whole brain. The Z-value standardized measures were spatially normalized to the MNI standard space (2 × 2× 2mm), and, finally, spatial smoothing with FWHM at 4mm^3^ was performed.

### Further calculated values

The fMRI measures ALFF, ReHo, and DC were extracted from all voxels of the triple network for each condition in all subjects using the binarized triple network masks. The extracted voxel-level values were used to calculate several parameters of interest, relevant for the examination of the task effect on the post task resting-state. These parameters and the exact description of how they were calculated are shown in Table 1.

**Table 1:** Description of parameters used to examine the effect of the task on the fMRI measures in the post-task RS.

Parameter	Calculation procedure/ meaning
**R1**	Voxel-level fMRI measures during the first (pre-task) resting-state (RS) (baseline)
**R2**	Voxel-level fMRI measures during the second (post-task) RS.
**T1, T2, T3**	Voxel-level fMRI measures during the three subtasks of the visual oddball paradigm.
**RS difference (RSD)**	Difference between post- and pre-task RS (R2—R1) in the voxel-level fMRI measures for each subject.
**Task**_**(whole)**_	Task _(whole)_ = (T1 + T2 +T3) / 3 (mean values of the fMRI measures during the three subtasks)
**Main task**_**(whole)**_	Task _(whole)_—R1
**RS similarity (RSS)**	Correlation coefficient between R1 and R2 for each subject.
**Task effect at the group level**	Correlation coefficients between the differences (Task (whole)–R1) and (R2—R1). All correlation coefficients were computed using Pearson’s correlation coefficients at a significance level of p < 0.05.

To compare the dynamics of the three fMRI measures during the different study conditions in the three networks, a three-way repeated measures analysis of variance (rm ANOVA) was carried out in order to investigate the effects of the network (three levels—DMN, CEN and SN), fMRI measure (ALFF, ReHo, DC), condition (R1, T1, T2, T3, R2) as well as the network*measure*condition interaction. Only in case of a statistically significant effect obtained in the global F test and a significant measure*condition interaction in the network, post hoc t tests were performed for multiple pairwise comparisons. Due to the different number of voxels in the networks and the whole-network based approach in our study, the mean values from the whole network for each of the three parameters were included in this analysis for each subject The calculations were performed using the software package IBM SPSS Version 25.

The inter-network FC of the three networks were calculated by first extracting the mean of the BOLD signal time series from the binarized mask of each network, followed by the computation of the Pearson’s correlation coefficient between each pair of networks. Fisher r to z transformation was performed to improve the normal distribution.

To investigate the relationship between the behavioral data (e.g. reaction time) and the fMRI measures, the correlation coefficients between the RSD and subject’s reaction time in the response condition was performed. Further, we investigated the association between the RT and the and the inter-network connectivity by calculating the correlation coefficients between the RT in the response condition and the inter-network connectivity. Also, to examine the relationship between the functional connectivity measures (ReHo, and DC), the correlation coefficients between the ReHo and the DC were calculated in each condition for each network.

Due to the exploratory approach, no correction for multiple comparisons was performed in this study.

## Results

### Behavioural data

The mean reaction time of the respond condition was 477 ms (SD = 13).

### Imaging data—task data

The task data were initially analyzed and reported following the examination of the first 16 participants [51]. The current analysis includes an enlarged collective of test subjects (N = 21) and confirms previously reported findings. In summary, activation in regions associated with a response to visual stimulation (occipital cortex) for both the target and the frequent stimuli was observed during all three subtasks of the visual oddball paradigm. Both, the count and respond conditions differed significantly from the passive condition in a number of brain regions including the pre- and post-central gyri, regions of the parietal cortex and the middle and inferior frontal gyri. Compared to the count condition, the response contrast yielded significant differences in the parietal operculum, inferior parietal lobule, insula, anterior cingulate cortex, and the posterior cingulate cortex (PCC). The BOLD activation in response to target and frequent stimuli in the passive, count and respond condition are shown in the S1 Fig. Further, in the S2 Fig we show the tripled two-group differences for the first level contrast (target > frequent stimuli) which was calculated in a pairwise compression of the three task conditions.

### Imaging data -triple network resting-state data

**Fig 2:**
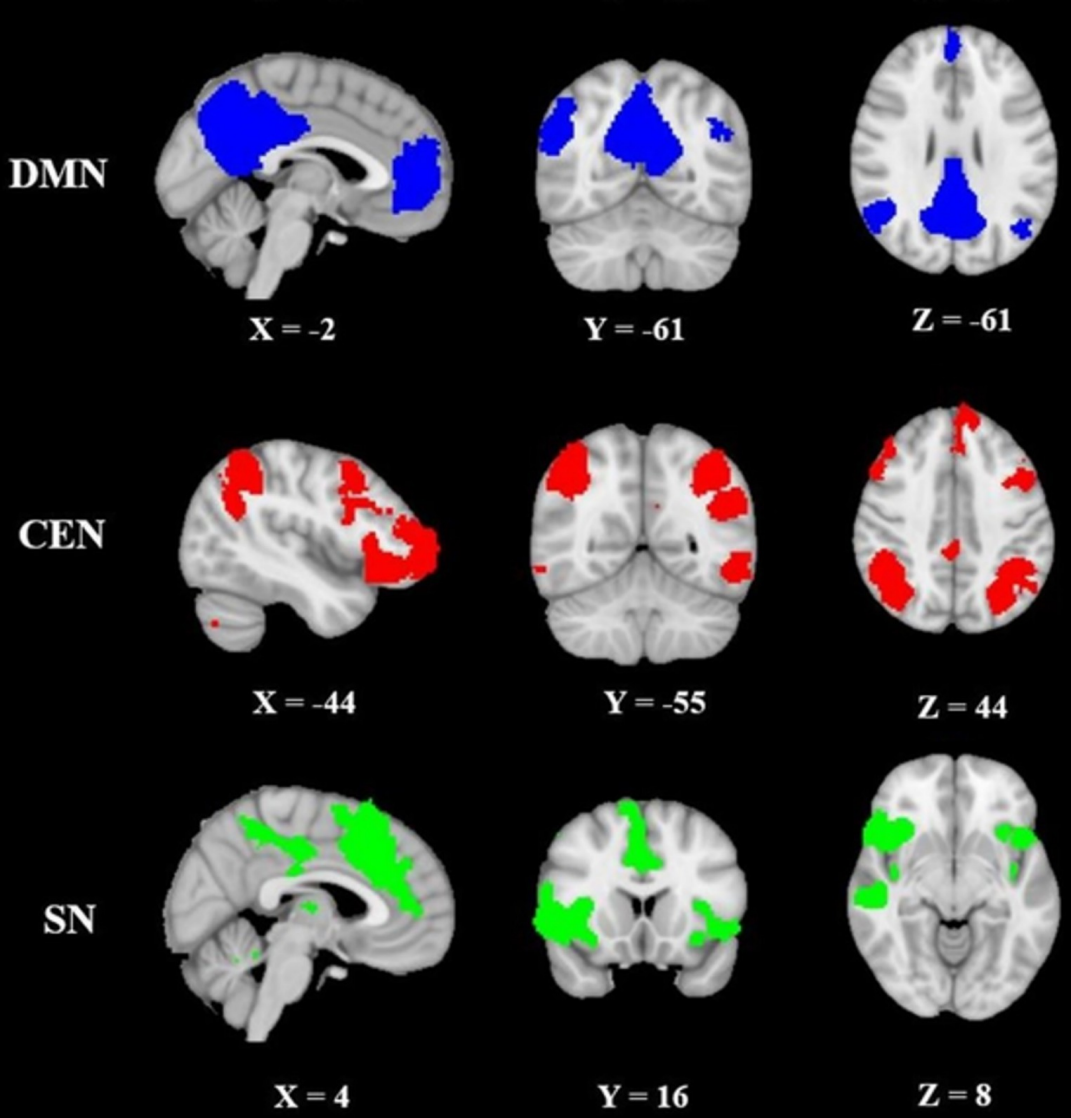
**Depiction of the triple networks referred to as the triple network: Default mode network (DMN, blue colour), central executive network (CEN, red colour), and salience network (SN, green colour).** The networks were identified by decomposing the pre-task resting-state condition into 20 components from 21 subjects.

The triple network was identified using group independent component analysis (Fig 2). Specifically, the DMN included the posterior cingulate cortex (PCC), precuneus, angular gyrus, and medial prefrontal cortex (mPFC); the CEN included the lateral posterior parietal cortex (LPPC) and dorsolateral prefrontal cortex (DLPFC); the SN included the frontal insular cortex (FIC), and anterior cingulate cortex (ACC).

### RSA and FC across different study conditions

The fMRI measures showed different values of the RSA and the local and global FC during the different study conditions (rest-task-rest). The mean values and the standard deviations of the fMRI measures during the two RS periods and the three subtasks based on the mean values of each parameter in 21 subjects are shown in Table 2.

**Table 2:** Mean values and standard deviations (SD) of the three fMRI measures during the two RS periods (R1 and R1) and the three subtasks of the VOP (T1, T2 and T3) obtained from the triple network (default mode network (DMN), salience network (SN), and central executive network (CEN)), based on the mean values of each parameter in 21 subjects.

Brain network		Task			
	R1 (mean (SD))	T1 (mean (SD))	T2 (mean (SD))	T3 (mean (SD))	R2 (mean (SD))
**Amplitude of low frequency fluctuations (ALFF)**					
DMN	0.042 (0.063)	0.046 (0.064)	0.044 (0.066)	0.048 (0.071)	0.047 (0.074)
CEN	-0.022 (0.063)	- 0.005 (0.058)	-0.005 (0.069)	-0.004 (0.068)	-0.011 (0.056)
SN	-0.012 (0.442)	-0.011 (0.051)	-0.019 (0.058)	-0.009 (0.058)	-0.018 (0.057)
**Regional homogeneity (ReHo)**					
DMN	0.375 (0.148)	0.419 (0.164)	0.373 (0.184)	0.404 (0.188)	0.393 (0.147)
CEN	0.265 (0.106)	0.319 (0.108)	0.323 (0.097)	0.334 (0.106)	0.261 (0.112)
SN	0.147 (0.126)	0.143 (0.118)	0.154 (0.111)	0.169 (0.133)	0.13 (0.103)
**Degree centrality (DC)**					
DMN	0.082 (0.099)	0.182 (0.225)	0.205 (0.283)	0.244 (0.308)	0.121 (0.116)
CEN	0.053 (0.079)	0.081 (0.159)	0.147 (0.191)	0.139 (0.187)	0.077 (0.116)
SN	0.040 (0.069)	0.024 (0.107)	0.044 (0.099)	0.061 (0.128)	0.043 (0.051)

**Fig 3:**
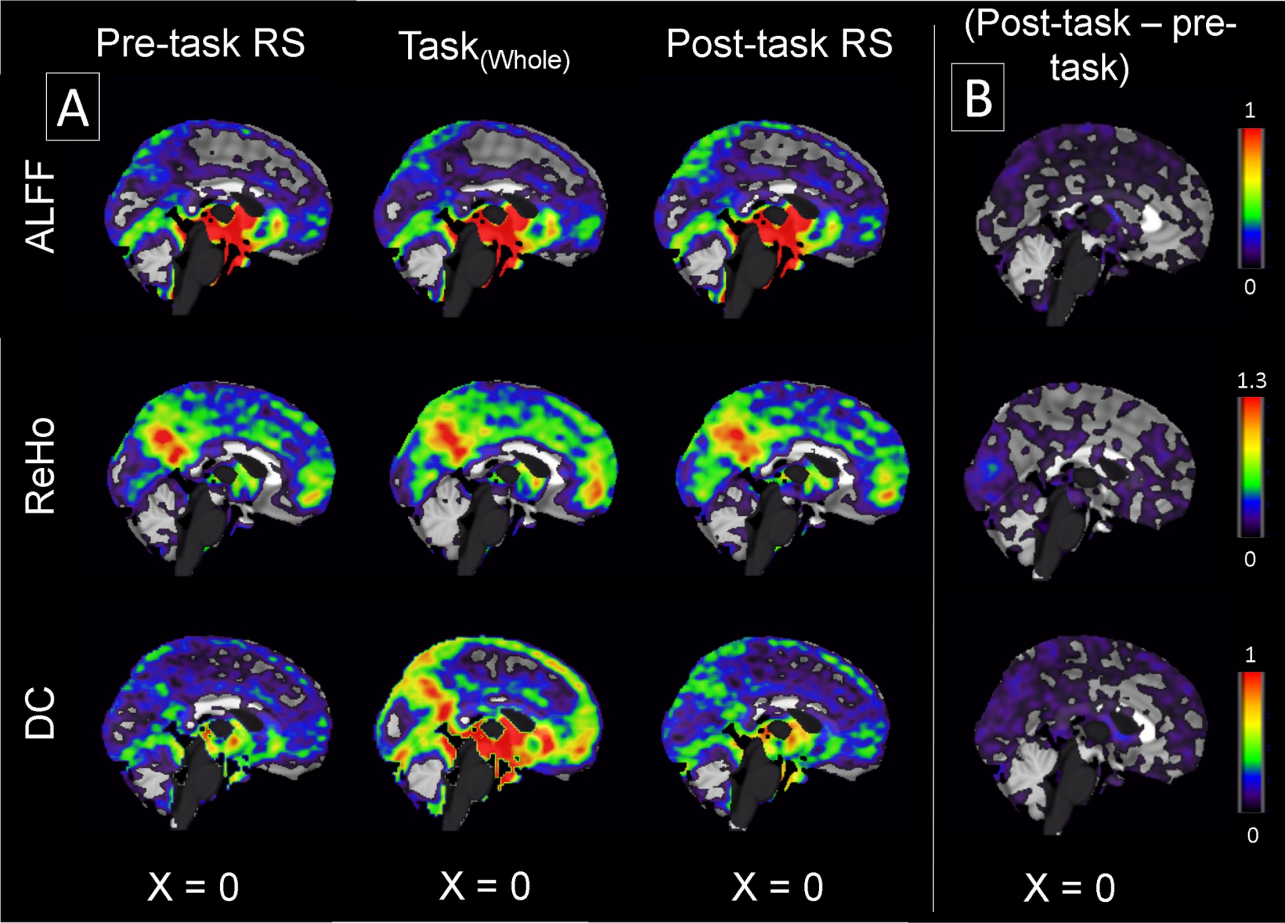
fMRI measures (ALFF, ReHo, and DC) from 21 subjects depicted for the whole brain. The difference between the two resting states (R2-R1) for each three fMRI measures is shown in the right row (B).

ALFF, ReHo and DC values obtained during the two resting states and during the whole task are depicted in Fig 3. The contrast between the two RS conditions (R2 –R1) is shown in the right part of the figure (Fig 3B).

**Fig 4:**
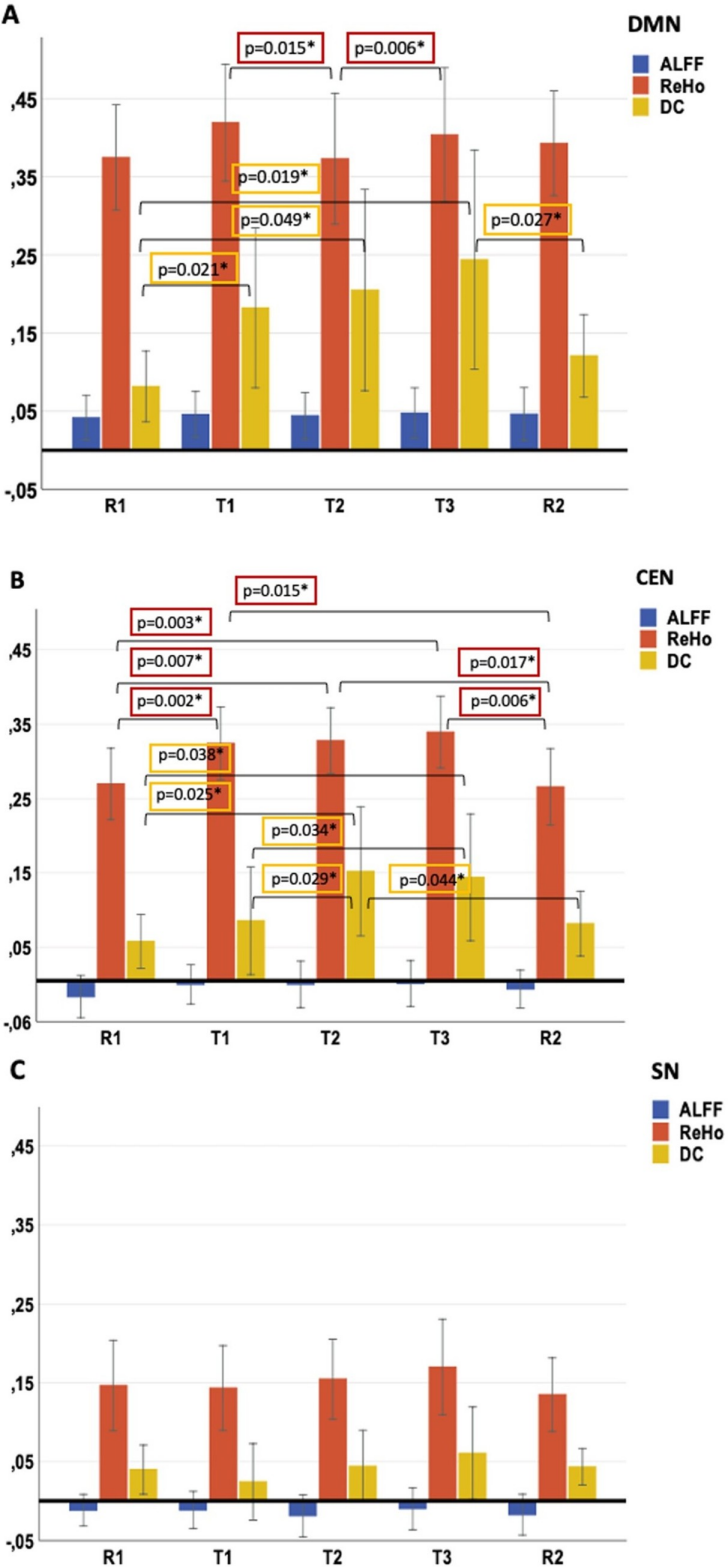
Bar chart representing the mean of the fMRI measures—amplitude of low-frequency fluctuations (ALFF), the regional homogeneity (ReHo), and the degree centrality (DC) in the three resting state networks—default mode network (DMN) (A), central executive network (CEN) (B) and salience network (SN) (C)- across 21 subjects in 5 conditions including resting-state 1 (R1), passive (T1), count (T2), respond (T3), and resting-state 2 (R2).

The dynamics of the changes in the individual fMRI parameters during the different phases of the study for the three core RS networks on which the focus of the current study was placed are shown in Fig 4. The three-way repeated measure ANOVA revealed a significant threefold interaction between the factors network, fMRI parameter and condition (F (5, 255) = 3.55; p = 0.005). Significant two way interactions could be observed for the parameters measure*condition within the DMN (F(2,752) = 4.355; p = 0.01) and for the CEN (F(4,115) = 2.953; p = 0.024). This interaction was not significant in the SN (F(4,835) = 0.803; p = 0.547). Thus, post hoc comparisons between the single condition for each fMRI measure were performed only in the DMN and CEN.

Due a significant Mauchly-test all reported values were adapted using the Greenhouse-Geisser method [64]. The post-hoc pair-wise comparation between the single values within the networks revealed some significant results as shown in Fig 4.

Concretely, we could not observe significant changes of the ALFF between the five investigated conditions in none of the three investigated core RS networks.

In the DMN the parameter ReHo increased non-significantly in T1 compared with R1. This value further decreased significantly during T2 (p = 0.015) and increased again significantly during T3 (p = 0.006). In the same network, the parameter DC increased significantly during T1 (p = 0.021) and remained significantly higher than in R1 during the both consecutive parts of the VOP (T2 (p = 0.049) and T3 (p = 0.019)). In the further course of the study, it decreased significantly in R2 (p = 0.027).

In the CEN, the parameter ReHo increased significantly during the task compared with R1 (T1: p = 0.002; T2: p = 0.007, T3: p = 0.002) and decreased significantly during the R2 (p = 0.006). Similar dynamics could be observed for the parameter DC, although the value did not increase significantly during the passive task condition but only in the T2 condition (p = 0.029) and then even further in the context of the T3 (p = 0.034). The value decreased to a level comparable with R1 in the R2 condition (p = 0.044).

Since these post hoc analyses were not confirmatory but rather merely exploratory, no correction for multiple testing was carried out.

### Inter-network interaction

**Fig 5:**
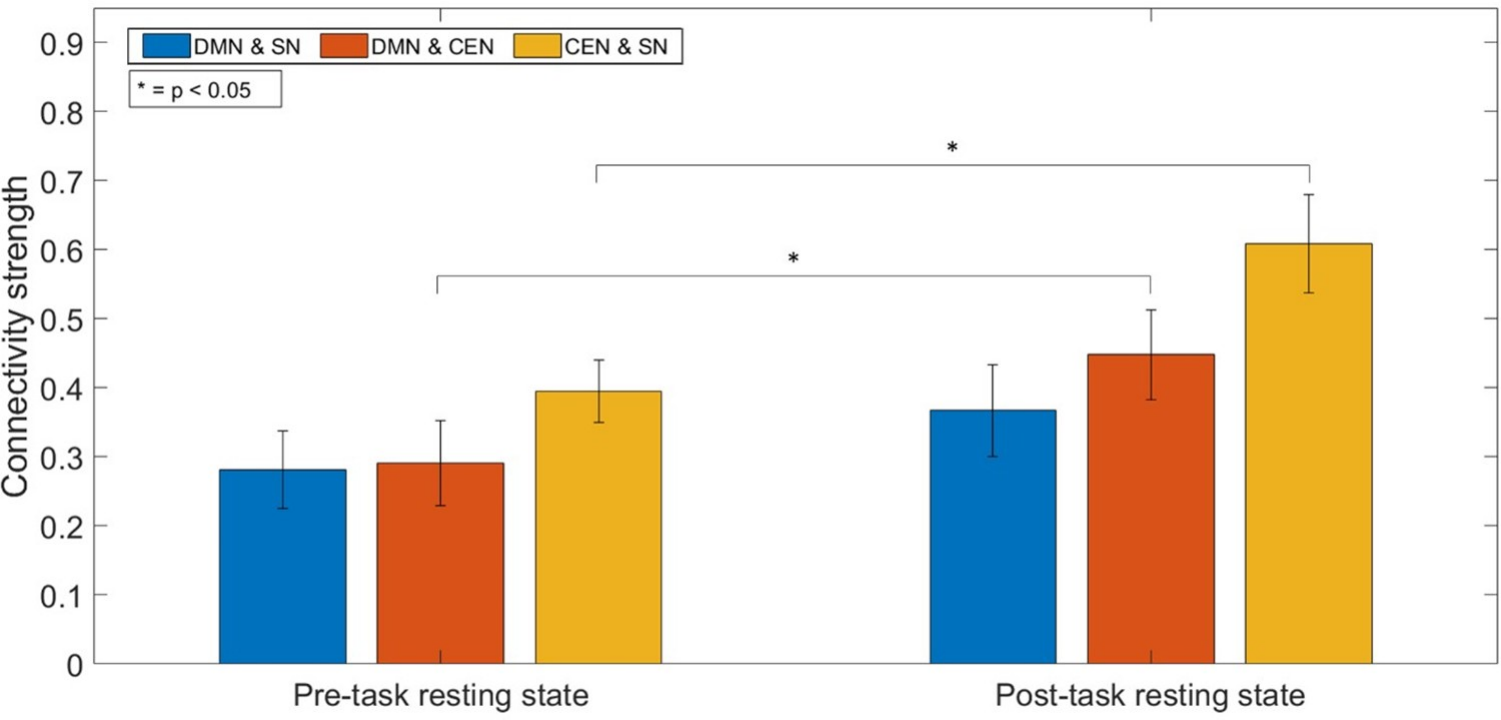
Strength of the FC between each pair of networks in the triple network in the pre- and post-task resting-state. There is a significant increase in FC between the DMN and CEN, and between the CEN and the SN in the post-task resting-state (p < 0.05). The bars represent the standard error.

The functional connectivity between the DMN and CEN increased significantly following the performance of the task (p = 0.015). The connectivity strength between the DMN and the SN remained stable (p = 0.25), whereas it increased significantly between the SN and CEN (p = 0.0004) (Fig 5).

The investigation of the relationship between the RT in the response condition and the inter-network connectivity revealed only a significant negative correlation between the RT and the inter-network connectivity between DMN and CEN (r = - 0.43, p = 0.04) in the pre-task RS (R1). All other correlations were not significant. Even more, the analysis of the associations between the RT and the inter-network connectivity changes remained also non-significant for all observations (change of DMN-SN inter-network connectivity and RT: r = -0.203; p = 0.377; change of DMN-CEN inter-network connectivity and RT: r = 0.252; p = 0.270; change of CEN-SN inter-network connectivity and RT: r = -0.165; p = 0.476).

### Associations between the pre- and post-task resting-state differences and the task

**Fig 6:**
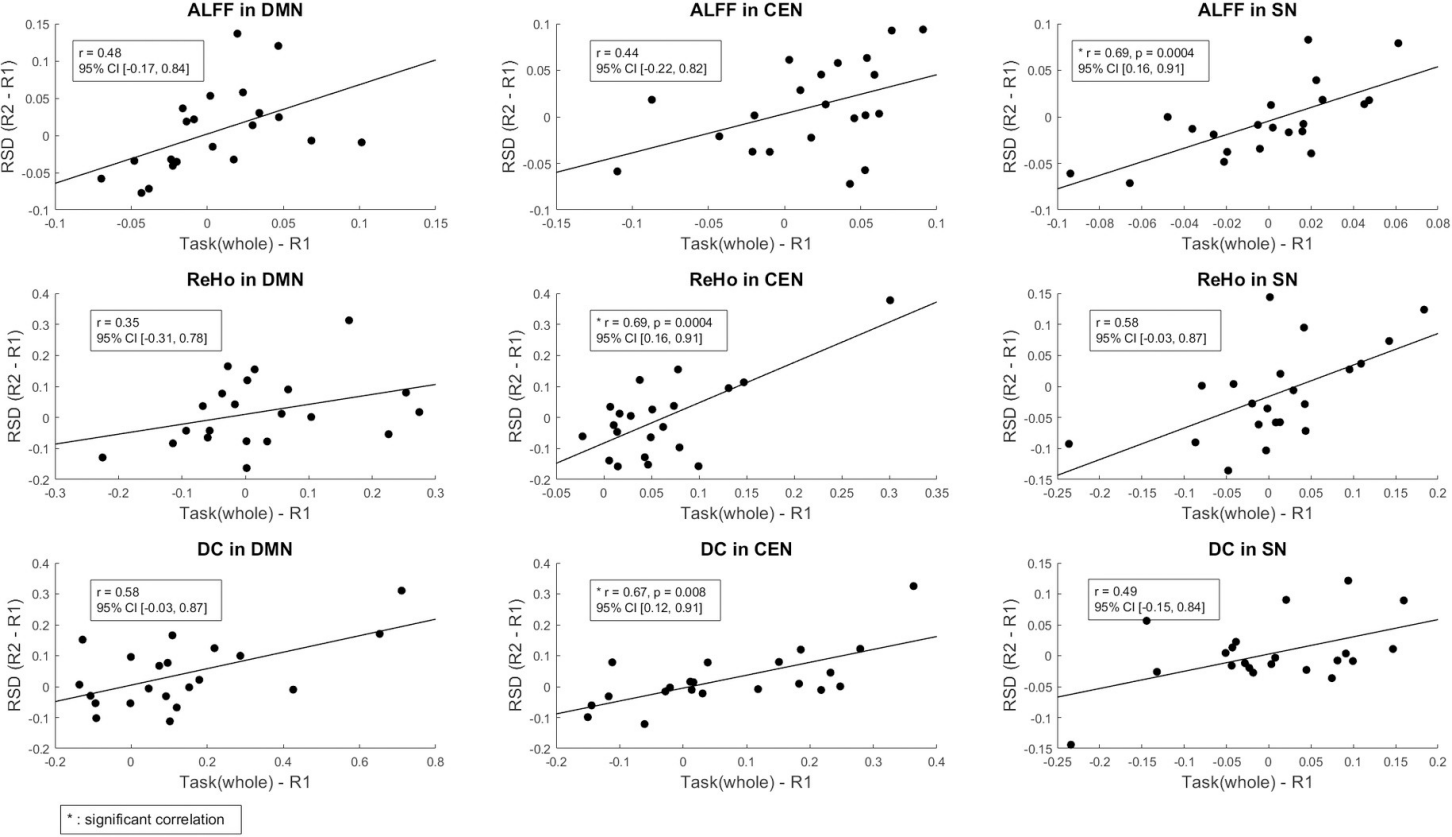
Correlations between the fMRI measures resulting from the pure task_(whole)_ effects and the RSD in the triple network, including the DMN, CEN, and SN of the fMRI measures.

The correlation between the differences between post-task and pre-task RS parameters (RSD = R2—R1) and the fMRI measures resulting from the pure task effects (task _(Whole)_—R1) are depicted in (Fig 6). Significant positive correlations were found in DMN for ALFF (r = 0.48, p = 0.02; 95% CI [-0.17, 0.84]) and DC (r = 0.58, p = 0.005; 95% CI [-0.03, 0.87]); in CEN for ALFF (r = 0.44, p = 0.04; 95% CI [-0.22, 0.82]), ReHo (r = 0.69, p = 0.004; 95% CI [0.16, 0.91]) and DC (r = 0.67, p = 0.008; 95% CI [0.12, 0.91]); and in SN for ALFF (r = 0.69, p = 0.004; 95% CI [0.16, 0.91]), ReHo (r = 0.58, p = 0.004; 95% CI [-0.03, 0.87]), and DC (r = 0.49, p = 0.02; 95% CI [-0.15, 0.84]). Taking into account the confidence intervals, only the correlations for ReHo and DC in CEN, and for ALFF in SN can be considered as significant.

The investigation of the associations between the RS differences (RSD and the subjects´ RT in the respond condition remained without significant correlations.

### Similarity between the first and the second RS and the associations between ReHo and DC

The RSS values calculated separately for the triple networks (DMN, CEN and SN) for each of the fMRI measures (ALFF; ReHo and DC) are shown in Table 3. The RSS values were comparable for all three parameters across all three networks.

**Table 3:** Mean values, standard deviation, and the range of the resting-state similarity (RSS) calculated separately for each resting-state fMRI parameter (ALFF, ReHo, and DC) and for each of the triple networks (default mode network (DMN), salience network (SN), and central executive network (CEN).

	RSS (Mean)	RSS (SD)	RSS (Range)
DMN			
ALFF	0.882	0.029	0.824–0.925
ReHo	0.812	0.039	0.698–0.876
DC	0.756	0.0657	0.575–0.884
CEN			
ALFF	0.874	0.044	0.763–0.946
ReHo	0.779	0.051	0.655–0.867
DC	0.757	0.097	0.425–0.864
SN			
ALFF	0.886	0.048	0.721–0.934
ReHo	0.758	0.045	0.664–0.851
DC	0.756	0.083	0.498–0.880

The results of the correlations between the two functional connectivity measures—ReHo and DC—remained highly significant in each condition of the investigation, as shown in Table 4. Furthermore, in all three networks we found a significant correlation between the extent of the change in the ReHo parameter (R2-R1) and the DC (R2-R1) parameter: in the DMN: r = 0.735, p < 0.001, in the CEN: r = 0.709, p < 0.001, in the SN: r = 0.586, p = 0.005.

**Table 4:** The relationships between the fMRI measures (ReHo, and DC) across the brain networks (DMN, CEN, and SN). All correlations coefficients were positive and significant at p < 0.01.

Correlations between ReHo and DC									
DMN									
R1		T1		T2		T3		R2	
r	p-value	r	p-value	r	p-value	r	p-value	r	p-value
0.88	< 0.01	0.82	< 0.01	0.84	< 0.01	0.77	< 0.01	0.78	< 0.01
CEN									
R1		T1		T2		T3		R2	
r	p-value	r	p-value	r	p-value	r	p-value	r	p-value
0.79	< 0.01	0.74	< 0.01	0.66	< 0.01	0.76	< 0.01	0.84	< 0.01
SN									
R1		T1		T2		T3		R2	
r	p-value	r	p-value	r	p-value	r	p-value	r	p-value
0.76	< 0.01	0.81	< 0.01	0.6	< 0.01	0.73	< 0.01	0.7	< 0.01

## Discussion

In this study, we investigated the effects of a simple visual-oddball paradigm on three basic fMRI measurements of the RS–ALFF (RSA), ReHo, and DC (the local and the global functional connectivity, respectively)—in the three core RS networks—DMN, CEN, and SN. Our analysis revealed different dynamics of changes in brain activity and connectivity in the three networks during the rest-task-rest investigation, the course of which was related to the different requirements of the individual subtasks. The assessment of these properties in the post-task RS phase, on the other hand, showed a high degree of similarity with the pre-task RS. Furthermore, the task performance induced a significant increase in the inter-network correlations between the SN and CEN, as well as between the DMN and CEN, but not between the DMN and SN. Also, the differences between the pre- and the post-task RS (R2—R1) were strongly associated with the main task influence (task _(Whole)_—R1) in two networks (for ReHo and DC in the CEN and for ALFF in the SN). Finally, at a behavioral level, the task performance (subject’s reaction time in the respond condition) correlated solely with the inter-network connectivity between DMN and CEN in the pre-task RS.

One particularity of our study was the investigation of the properties of the core resting state networks addressing the whole networks, rather the single subregions. In this approach, the temporal dynamic of the intrinsic brain networks is more robust compared to the analysis of single subregions. This is due to a lower susceptibility to noises caused by head motion, respiration, and vascular pulsatility [65,66]. Furthermore, this approach allows a statement to be made about the overall function of the networks, rather than the function of single subregions, which in most cases cannot be assigned exclusively to one network. Therefore, our approach considered for each subject the mean values of the voxel-wise fMRI measurements as representative of the properties of the individual networks. These values were used to capture the dynamics of task-induced changes in the individual networks.

Interestingly, we could not find a significant change in the ALFF values at the network level in none of the three investigated networks. This is a somewhat unexpected result, especially since the DMN deactivation during task performance has been reported in numerous studies using different neuroimaging techniques [67–69]. Thereby, it is hypothesized that the deactivation results from reallocation of attentional resources from internal to external sources of information [69]. However, the deactivation extent appears to be associated with the cognitive demand of the task: persisted activity of the DMN is been reported during not sufficiently challenging tasks [7] as well as a lower extent of DMN deactivation has been shown during decreased task demands [70,71]. Indeed, the VOP, particularly the passive subtask, includes a very low cognitive demand limited on a passive observation of the presented stimuli. In our study, the passive stimuli observation resulted mainly in the activation of the visual cortex for both, the target and the frequent stimuli, which could be observed during all three conditions. Although the general activation was much more extensive in the two other subtasks, we did not observe any significant change in ALFF values in the three target networks in any of these conditions. In one previous investigation, the authors reported a reduction of both, fractional ALFF (fALFF) and FC during sustained working memory task in the posterior areas of DMN, while changes in the ACC were less marked [72]. In contrast to this study, our approach considers the entire network, so that the lack of significant changes in ALFF values could be attributed to a cumulative mutual cancellation of the different changes in different parts of the network.

During the subtask T2, however, in which not only passive observation but also active counting of irregular stimuli was required, we observed a significant reduction in the ReHo value in DMN, which is in concordance with previously reported reduction of ReHo in the DMN during a simple cognitive task [48]. Generally, a reduction in ReHo seems to indicate a decrease in activity in the observed region [73,74]. Interestingly, a renewed increase in ReHo occurred in the DMN during the response task (T3). ReHo, often referred to as the local FC is defined by the temporal coherence or synchronization of the BOLD time series within a set of a given voxel’s nearest neighbors [26]. ReHo represents the most efficient, reliable, and widely used index of local FC [75,76]. An increase in ReHo indicates an increased local synchronization of spontaneous neural activity [72]. This finding may be attributed to the fact, that an important part of the DMN–the posterior cingulate cortex (PCC)—showed a significant activation in the respond condition only.

The described alterations were accompanied by an incremental increase in DC values and their significant reduction after the transition to the second RS. DC represents the overall connectivity between particular brain regions to other brain areas [8,75]. An increase in DC of specific brain hubs indicates a more extensive communication with distinct brain areas. Earlier investigations reveal that the increase of functional connectivity may result in higher global efficiency that facilitates global information transmission [77] that has been required during the tasks performance. Otherwise, a DC decrease after task performance has been previously reported after subjects performed a sustained auditory working memory task [72].

In the CEN, we observed a gradual increase in ReHo in DC during the task and a significant decrease of both values and thus a return to the level observed during R1 after the completion of the task. At a broad level, the CEN is included in higher order executive functioning, including the cognitive control of thought, emotion regulation, and working memory [16,78,79] and is thus activated during efforts to exert self-control, reappraise threatening stimuli, and to suppress intrusive, unpleasant thoughts [80–82]. CEN activity has been shown to be anti-correlated with activity in the DMN in healthy adults [1,18,19], while some investigations indicate that the CEN also exhibits an inhibitory control on the DMN [83]. Thus, the task associated ReHo and DC increase in the CEN express the involvement of this network in responding to the task demands. The decrease following the completion of a cognitive paradigm may be the basis for the restoration of the regular activity of the DMN within the scope of a decline in DMN inhibition which occurred as a result of increased CEN activity during the task performance.

All three investigated fMRI measures remained broadly stable during the rest-task-rest design in the SN. The SN as a network known to demonstrate competitive interactions during cognitive information processing [6,19] and, thus, having a critical role in switching between two other major RS networks (the DMN and the CEN [1]. In particular, the main hubs of the SN, the frontal inferior insula and ACC, are known to share significant topographic reciprocal connectivity and form a tightly coupled network, ideally placed to integrate information from several brain regions [84,85]. Thus, they seem to moderate arousal during cognitively demanding tasks and play a unique function in initiating control signals that activate the CEN and deactivate the DMN [18]. Previous investigations have linked increased ALFF values in some parts of the SN to a hyperarousal state in patients with MDD [86]. The stabile levels of RSA and FC before, during and after a low demanding task in healthy controls could reflect a lack of significant change in arousal by a task that neither required significant cognitive effort from participants nor had any affective components.

The connectivity analysis between the three networks revealed an increased synchronization (in terms of a significantly increased connectivity strength) for the SN with the CEN but not with the DMN in the post-task RS compared to the pre-task RS. This may be an after-task effect of the inter-network interactions during the paradigm performance. Indeed, Sridharan and colleagues have shown that the connectivity strength during the visual oddball paradigm particularly increased between the main nodes of the SN (frontal anterior insula and ACC) and all main nodes of the CEN, while the interactions between the SN and DMN were less pronounced [18].

Interestingly, the connectivity between the CEN and DMN also increased in the post-task resting-state. This finding is consistent with the literature on the cooperative activity of the DMN and the CEN during different mental operations [87]. An increased coupling between some parts of these two networks has been shown in problem-solving tasks [88], social working memory [89], and during creative idea production [90]. Furthermore, a significant interaction between the DMN and the CEN has also been shown during the RS condition [91].

In our study we observed an association between a stronger pre-task interaction between this two networks and better task performance (expressed here through shorter reaction times). Better synchronisation of the two networks at rest therefore seems to form a basis for faster responsiveness. Thereby, this interaction seems to fluctuate dynamically across short time scales [92], indicating that the temporal relationships between the DMN and CEN shifts depending on the change in the attention focus and the immediately preceding activity. Thus, the increased connectivity between the DMN and the CEN in the post-task RS observed in our study may be an expression of the shifting of attention after task completion.

Several subregions of the triple networks are known to be activated during the performance of cognitively demanding tasks [93]. In the case of the VOP performed in our study, the main task specific activation has been reported previously by Warbrick and colleagues [51]. The target detection specifically activated distinct brain regions than the frequent stimuli during the count and response condition in several brain regions including parietal and frontal regions (partly included in the CEN), with more widespread, left hemisphere dominant pre and post central gyri and frontal pole activation for the respond condition [51]. During the last part of the paradigm (T3), the target>frequent stimuli contrast mainly involved, besides the mentioned parts of the CEN, also parts of the DMN (PCC) and the SN (Insula, ACC). The insula activation was common to the count and respond conditions. The intensive involvement of different subregions of the triple networks in the performance of the task may have contributed to the dynamics in the triple network model networks during this rest-task-rest design. Indeed, we observed positive correlations between the extent of the differences between R1 and R2 regarding specific parameters and the actual task effect on the same parameters in the triple networks. These correlations were significant in the CEN for ReHo and DC measures and in the SN for ALFF. A close relationship between the cognitive level of the previous task and the extent of the modulation in the brain networks has been reported previously. Barnes and colleagues observed that the changes in endogenous dynamics in post-task RS is directly related to the difficulty of task performance [94]. In the case of the VOP used here, the levels of cognitive demand for all three subtasks are not widely different and the whole paradigm did not require high cognitive effort. Accordingly, we observed a high level of resting state similarity for all three parameters in all three networks. However, we observed an increased synchronization for the CEN with the SN as well as with the DMN in the post-task RS. This may be an expression on a more intensive involvement of several subregions of the CEN in the last two parts of the paradigm. Even more, the T3 subtask also included a pronounced activation of the insula and the ACC (parts of the SN) as well as the PCC (DMN) during the counting and responding to the target. Thus, the increased synchronization of the CEN with the SN as well as with the DMN in the post-task RS may be a consequence of this immediately preceding joint activity. Indeed, the extent of the changes in the RSA and local as well as global connectivity in the core RS networks in the post-task condition followed the extent of the task-induced changes within those networks.

## Conclusion

In conclusion, in this work we report the dynamics of changes in RSA, local and global connectivity within the triple RS networks during a simple cognitive activity. A particularity of this work is the capture of the properties of the networks as a whole. The observed patterns of change differed noticeably between the networks and were tightly associated with the task-related brain activity and the distinct involvement of the networks in the performance of the different subtasks. In the post-task RS we could find a very high similarity with the pre-task RS in the individual parameters within the individual networks. However, especially the inter-network analysis showed an increased synchronization of CEN with the DMN and the SN after the task. The differences between the pre- and post-task RS correlated largely with the extent of the pure task-effect. Furthermore, we observed that the RT was shorter (and thus the performance better) in the case of a higher pre-task interaction between the DMN and the CEN. In sum, our results show that the behavior/task not only led to dynamic changes in the properties of three core resting state networks, but also to a change in the interactions between the networks, which could be detected at least immediately after the task. This in turn could have an impact on subsequent activities.

Our results provide some additional insight into the task associated dynamics within and between the triple resting state networks. Further investigations are required in order to understand better their functional importance and interplay.

## Supporting information

S1 Fig BOLD activation in response to target and frequent stimuli. Z: Cluster-corrected threshold.
(DOCX)

S2 Fig BOLD activation of the tripled two-group difference (“tripled t-test”). Z: Cluster-corrected threshold.
(DOCX)

## References

[1] Menon V, Uddin LQ. Saliency, switching, attention and control: a network model of insula function. Brain Struct Funct. 2010 May 29;214(5–6):655–67. doi: 10.1007/s00429-010-0262-0 20512370 PMC2899886

[2] Menon V. Large-scale brain networks and psychopathology: a unifying triple network model. Trends in Cognitive Sciences. 2011 Oct;15(10):483–506. doi: 10.1016/j.tics.2011.08.003 21908230

[3] Wu X, Li Q, Yu X, Chen K, Fleisher AS, Guo X, et al. A Triple Network Connectivity Study of Large-Scale Brain Systems in Cognitively Normal APOE4 Carriers. Front Aging Neurosci. 2016 Sep 28;8. doi: 10.3389/fnagi.2016.00231 27733827 PMC5039208

[4] Krishnadas R, Ryali S, Chen T, Uddin L, Supekar K, Palaniyappan L, et al. Resting state functional hyperconnectivity within a triple network model in paranoid schizophrenia. The Lancet. 2014 Feb;383:S65. doi: 10.1016/S0140-6736(14)60328-7

[5] Lin P, Yang Y, Gao J, De Pisapia N, Ge S, Wang X, et al. Dynamic Default Mode Network across Different Brain States. Sci Rep. 2017 Apr 6;7(1). doi: 10.1038/srep46088 28382944 PMC5382672

[6] Greicius MD, Krasnow B, Reiss AL, Menon V. Functional connectivity in the resting brain: A network analysis of the default mode hypothesis. Proceedings of the National Academy of Sciences. 2002 Dec 27;100(1):253–8. doi: 10.1073/pnas.0135058100 12506194 PMC140943

[7] Greicius MD, Menon V. Default-Mode Activity during a Passive Sensory Task: Uncoupled from Deactivation but Impacting Activation. Journal of Cognitive Neuroscience. 2004 Nov;16(9):1484–92. doi: 10.1162/0898929042568532 15601513

[8] van den Heuvel MP, Sporns O. Network hubs in the human brain. Trends in Cognitive Sciences. 2013 Dec;17(12):683–96. doi: 10.1016/j.tics.2013.09.012 24231140

[9] Hagmann P, Cammoun L, Gigandet X, Meuli R, Honey CJ, Wedeen VJ, et al. Mapping the Structural Core of Human Cerebral Cortex. Friston KJ, editor. PLoS Biol. 2008 Jul 1;6(7):e159. doi: 10.1371/journal.pbio.0060159 18597554 PMC2443193

[10] Elton A, Gao W. Task-positive Functional Connectivity of the Default Mode Network Transcends Task Domain. Journal of Cognitive Neuroscience. 2015 Dec;27(12):2369–81. doi: 10.1162/jocn_a_00859 26244722

[11] Wang Z, Liu J, Zhong N, Qin Y, Zhou H, Li K. Changes in the brain intrinsic organization in both on-task state and post-task resting state. NeuroImage. 2012 Aug;62(1):394–407. doi: 10.1016/j.neuroimage.2012.04.051 22569542

[12] Li B, Wang X, Yao S, Hu D, Friston K. Task-Dependent Modulation of Effective Connectivity within the Default Mode Network. Front Psychology. 2012;3. doi: 10.3389/fpsyg.2012.00206 22737141 PMC3381220

[13] Müller NG, Knight RT. The functional neuroanatomy of working memory: Contributions of human brain lesion studies. Neuroscience. 2006 Apr;139(1):51–8. doi: 10.1016/j.neuroscience.2005.09.018 16352402

[14] Bressler SL, Menon V. Large-scale brain networks in cognition: emerging methods and principles. Trends in Cognitive Sciences. 2010 Jun;14(6):277–90. doi: 10.1016/j.tics.2010.04.004 20493761

[15] Li Q, Liu J, Wang W, Wang Y, Li W, Chen J, et al. Disrupted coupling of large-scale networks is associated with relapse behaviour in heroin-dependent men. jpn. 2018 Jan 1;43(1):48–57. doi: 10.1503/jpn.170011 29252165 PMC5747535

[16] Seeley WW, Menon V, Schatzberg AF, Keller J, Glover GH, Kenna H, et al. Dissociable Intrinsic Connectivity Networks for Salience Processing and Executive Control. Journal of Neuroscience. 2007 Feb 28;27(9):2349–56. doi: 10.1523/JNEUROSCI.5587-06.2007 17329432 PMC2680293

[17] Goulden N, Khusnulina A, Davis NJ, Bracewell RM, Bokde AL, McNulty JP, et al. The salience network is responsible for switching between the default mode network and the central executive network: Replication from DCM. NeuroImage]. 2014 Oct;99:180–90. doi: 10.1016/j.neuroimage.2014.05.052 24862074

[18] Sridharan D, Levitin DJ, Menon V. A critical role for the right fronto-insular cortex in switching between central-executive and default-mode networks. Proceedings of the National Academy of Sciences. 2008 Aug 22;105(34):12569–74. doi: 10.1073/pnas.0800005105 18723676 PMC2527952

[19] Fox MD, Snyder AZ, Vincent JL, Corbetta M, Van Essen DC, Raichle ME. From The Cover: The human brain is intrinsically organized into dynamic, anticorrelated functional networks. Proceedings of the National Academy of Sciences. 2005 Jun 23;102(27):9673–8. doi: 10.1073/pnas.0504136102 15976020 PMC1157105

[20] Shannon BJ, Raichle ME, Snyder AZ, Fair DA, Mills KL, Zhang D, et al. Premotor functional connectivity predicts impulsivity in juvenile offenders. Proceedings of the National Academy of Sciences. 2011 Jun 27;108(27):11241–5. doi: 10.1073/pnas.1108241108 21709236 PMC3131347

[21] Chand GB, Dhamala M. Interactions Among the Brain Default-Mode, Salience, and Central-Executive Networks During Perceptual Decision-Making of Moving Dots. Brain Connectivity. 2016 Apr;6(3):249–54. doi: 10.1089/brain.2015.0379 26694702

[22] Zheng H, Xu L, Xie F, Guo X, Zhang J, Yao L, et al. The Altered Triple Networks Interaction in Depression under Resting State Based on Graph Theory. BioMed Research International [Internet]. 2015;2015:1–8. doi: 10.1155/2015/386326 26180798 PMC4477135

[23] Liu Y, Li L, Li B, Feng N, Li L, Zhang X, et al. Decreased Triple Network Connectivity in Patients with Recent Onset Post-Traumatic Stress Disorder after a Single Prolonged Trauma Exposure. Sci Rep. 2017 Oct 3;7(1). doi: 10.1038/s41598-017-12964-6 28974724 PMC5626705

[24] Fan J, Zhong M, Gan J, Liu W, Niu C, Liao H, et al. Altered connectivity within and between the default mode, central executive, and salience networks in obsessive-compulsive disorder. Journal of Affective Disorders. 2017 Dec;223:106–14. doi: 10.1016/j.jad.2017.07.041 28743059

[25] Manoliu A, Riedl V, Zherdin A, Mühlau M, Schwerthöffer D, Scherr M, et al. Aberrant Dependence of Default Mode/Central Executive Network Interactions on Anterior Insular Salience Network Activity in Schizophrenia. Schizophrenia Bulletin. 2013 Mar 21;40(2):428–37. doi: 10.1093/schbul/sbt037 23519021 PMC3932085

[26] Jiang L, Zuo X-N. Regional Homogeneity. Neuroscientist. 2016 Jul 9;22(5):486–505. doi: 10.1177/1073858415595004 26170004 PMC5021216

[27] Menon B. Towards a new model of understanding–The triple network, psychopathology and the structure of the mind. Medical Hypotheses. 2019 Dec;133:109385. doi: 10.1016/j.mehy.2019.109385 31494485

[28] Imperatori C, Massullo C, Carbone GA, Panno A, Giacchini M, Capriotti C, et al. Increased Resting State Triple Network Functional Connectivity in Undergraduate Problematic Cannabis Users: A Preliminary EEG Coherence Study. Brain Sciences. 2020 Feb 28;10(3):136. doi: 10.3390/brainsci10030136 32121183 PMC7139645

[29] Sakoğlu Ü, Pearlson GD, Kiehl KA, Wang YM, Michael AM, Calhoun VD. A method for evaluating dynamic functional network connectivity and task-modulation: application to schizophrenia. Magn Reson Mater Phy. 2010 Feb 17;23(5–6):351–66. doi: 10.1007/s10334-010-0197-8 20162320 PMC2891285

[30] Bassett DS, Wymbs NF, Porter MA, Mucha PJ, Carlson JM, Grafton ST. Dynamic reconfiguration of human brain networks during learning. Proceedings of the National Academy of Sciences [Internet]. 2011 Apr 18;108(18):7641–6. doi: 10.1073/pnas.1018985108 21502525 PMC3088578

[31] Albert NB, Robertson EM, Miall RC. The Resting Human Brain and Motor Learning. Current Biology. 2009 Jun;19(12):1023–7. doi: 10.1016/j.cub.2009.04.028 19427210 PMC2701987

[32] Lewis CM, Baldassarre A, Committeri G, Romani GL, Corbetta M. Learning sculpts the spontaneous activity of the resting human brain. Proceedings of the National Academy of Sciences. 2009 Oct 5;106(41):17558–63. doi: 10.1073/pnas.0902455106 19805061 PMC2762683

[33] Stevens WD, Buckner RL, Schacter DL. Correlated Low-Frequency BOLD Fluctuations in the Resting Human Brain Are Modulated by Recent Experience in Category-Preferential Visual Regions. Cerebral Cortex. 2009 Dec 21;20(8):1997–2006. doi: 10.1093/cercor/bhp270 20026486 PMC2901023

[34] Taubert M, Lohmann G, Margulies DS, Villringer A, Ragert P. Long-term effects of motor training on resting-state networks and underlying brain structure. NeuroImage. 2011 Aug;57(4):1492–8. doi: 10.1016/j.neuroimage.2011.05.078 21672633

[35] Sami S, Robertson EM, Miall RC. The Time Course of Task-Specific Memory Consolidation Effects in Resting State Networks. Journal of Neuroscience. 2014 Mar 12;34(11):3982–92. doi: 10.1523/JNEUROSCI.4341-13.2014 24623776 PMC3951697

[36] Hartzell JF, Tobia MJ, Davis B, Cashdollar NM, Hasson U. Differential lateralization of hippocampal connectivity reflects features of recent context and ongoing demands: An examination of immediate post-task activity. Hum Brain Mapp. 2014 Oct 8;36(2):519–37. doi: 10.1002/hbm.22644 25293364 PMC6869213

[37] Gregory MD, Agam Y, Selvadurai C, Nagy A, Vangel M, Tucker M, et al. Resting state connectivity immediately following learning correlates with subsequent sleep-dependent enhancement of motor task performance. NeuroImage [Internet]. 2014 Nov;102:666–73. doi: 10.1016/j.neuroimage.2014.08.044 25173415 PMC4252600

[38] Squires NK, Squires KC, Hillyard SA. Two varieties of long-latency positive waves evoked by unpredictable auditory stimuli in man. Electroencephalography and Clinical Neurophysiology. 1975 Apr;38(4):387–401. doi: 10.1016/0013-4694(75)90263-1 46819

[39] Bledowski C. Localizing P300 Generators in Visual Target and Distractor Processing: A Combined Event-Related Potential and Functional Magnetic Resonance Imaging Study. Journal of Neuroscience. 2004 Oct 20;24(42):9353–60. doi: 10.1523/JNEUROSCI.1897-04.2004 15496671 PMC6730097

[40] Gur RC, Turetsky BI, Loughead J, Waxman J, Snyder W, Ragland JD, et al. Hemodynamic responses in neural circuitries for detection of visual target and novelty: An event-related fMRI study. Hum Brain Mapp. 2007;28(4):263–74. doi: 10.1002/hbm.20319 17133387 PMC6871418

[41] Kiehl KA, Stevens MC, Laurens KR, Pearlson G, Calhoun VD, Liddle PF. An adaptive reflexive processing model of neurocognitive function: supporting evidence from a large scale (n = 100) fMRI study of an auditory oddball task. NeuroImage. 2005 Apr;25(3):899–915. doi: 10.1016/j.neuroimage.2004.12.035 15808990

[42] Strobel A, Debener S, Sorger B, Peters JC, Kranczioch C, Hoechstetter K, et al. Novelty and target processing during an auditory novelty oddball: A simultaneous event-related potential and functional magnetic resonance imaging study. NeuroImage. 2008 Apr;40(2):869–83. doi: 10.1016/j.neuroimage.2007.10.065 18206391

[43] Warbrick T, Mobascher A, Brinkmeyer J, Musso F, Richter N, Stoecker T, et al. Single-trial P3 amplitude and latency informed event-related fMRI models yield different BOLD response patterns to a target detection task. NeuroImage. 2009 Oct;47(4):1532–44. doi: 10.1016/j.neuroimage.2009.05.082 19505583

[44] Linden DEJ. The Functional Neuroanatomy of Target Detection: An fMRI Study of Visual and Auditory Oddball Tasks. Cerebral Cortex. 1999 Dec 1;9(8):815–23. doi: 10.1093/cercor/9.8.815 10601000

[45] McCarthy G, Luby M, Gore J, Goldman-Rakic P. Infrequent Events Transiently Activate Human Prefrontal and Parietal Cortex as Measured by Functional MRI. Journal of Neurophysiology. 1997 Mar 1;77(3):1630–4. doi: 10.1152/jn.1997.77.3.1630 9084626

[46] Yoshiura T, Zhong J, Shibata DK, Kwok WE, Shrier DA, Numaguchi Y. Functional MRI study of auditory and visual oddball tasks. NeuroReport. 1999 Jun;10(8):1683–8. doi: 10.1097/00001756-199906030-00011 10501557

[47] Halgren E, Marinkovic K, Chauvel P. Generators of the late cognitive potentials in auditory and visual oddball tasks. Electroencephalography and Clinical Neurophysiology. 1998 Feb;106(2):156–64. doi: 10.1016/s0013-4694(97)00119-3 9741777

[48] Zang Y, Jiang T, Lu Y, He Y, Tian L. Regional homogeneity approach to fMRI data analysis. NeuroImage. 2004 May;22(1):394–400. doi: 10.1016/j.neuroimage.2003.12.030 15110032

[49] Zuo X-N, Ehmke R, Mennes M, Imperati D, Castellanos FX, Sporns O, et al. Network Centrality in the Human Functional Connectome. Cerebral Cortex. 2011 Oct 2;22(8):1862–75. doi: 10.1093/cercor/bhr269 21968567

[50] Yu-Feng Z, Yong H, Chao-Zhe Z, Qing-Jiu C, Man-Qiu S, Meng L, et al. Altered baseline brain activity in children with ADHD revealed by resting-state functional MRI. Brain and Development. 2007 Mar;29(2):83–91. doi: 10.1016/j.braindev.2006.07.002 16919409

[51] Warbrick T, Reske M, Shah NJ. Do EEG paradigms work in fMRI? Varying task demands in the visual oddball paradigm: Implications for task design and results interpretation. NeuroImage. 2013 Aug;77:177–85. doi: 10.1016/j.neuroimage.2013.03.026 23537937

[52] Smith SM. Fast robust automated brain extraction. Hum Brain Mapp. 2002 Nov;17(3):143–55. doi: 10.1002/hbm.10062 12391568 PMC6871816

[53] Jenkinson M, Bannister P, Brady M, Smith S. Improved Optimization for the Robust and Accurate Linear Registration and Motion Correction of Brain Images. NeuroImage. 2002 Oct;17(2):825–41. doi: 10.1016/s1053-8119(02)91132-8 12377157

[54] Jenkinson M, Smith S. A global optimisation method for robust affine registration of brain images. Medical Image Analysis. 2001 Jun;5(2):143–56. doi: 10.1016/s1361-8415(01)00036-6 11516708

[55] Shirer WR, Ryali S, Rykhlevskaia E, Menon V, Greicius MD. Decoding Subject-Driven Cognitive States with Whole-Brain Connectivity Patterns. Cerebral Cortex. 2011 May 26;22(1):158–65. doi: 10.1093/cercor/bhr099 21616982 PMC3236795

[56] Woolrich MW, Ripley BD, Brady M, Smith SM. Temporal Autocorrelation in Univariate Linear Modeling of FMRI Data. NeuroImage. 2001 Dec;14(6):1370–86. doi: 10.1006/nimg.2001.0931 11707093

[57] Woolrich MW, Behrens TEJ, Beckmann CF, Jenkinson M, Smith SM. Multilevel linear modelling for FMRI group analysis using Bayesian inference. NeuroImage. 2004 Apr;21(4):1732–47. doi: 10.1016/j.neuroimage.2003.12.023 15050594

[58] Yan C-G, Wang X-D, Zuo X-N, Zang Y-F. DPABI: Data Processing & Analysis for (Resting-State) Brain Imaging. Neuroinform. 2016 Apr 13;14(3):339–51. doi: 10.1007/s12021-016-9299-4 27075850

[59] Yan. DPARSF: a MATLAB toolbox for “pipeline” data analysis of resting-state fMRI. Front Syst Neurosci. 2010. doi: 10.3389/fnsys.2010.00013 20577591 PMC2889691

[60] Friston K. J., Williams S., Howard R., Frackowiak R. S. J., & Turner R. (1996). Movement-Related effects in fMRI time-series. Magnetic Resonance in Medicine, 35(3), 346–355. doi: 10.1002/mrm.1910350312 8699946

[61] Takeuchi H, Taki Y, Nouchi R, Sekiguchi A, Hashizume H, Sassa Y, et al. Degree centrality and fractional amplitude of low-frequency oscillations associated with Stroop interference. NeuroImage. 2015 Oct;119:197–209. doi: 10.1016/j.neuroimage.2015.06.058 26123381

[62] Gideon RA. The Correlation Coefficients. J Mod App Stat Meth. 2007 Nov 1;6(2):517–29. doi: 10.22237/jmasm/1193890500

[63] Zou Q-H, Zhu C-Z, Yang Y, Zuo X-N, Long X-Y, Cao Q-J, et al. An improved approach to detection of amplitude of low-frequency fluctuation (ALFF) for resting-state fMRI: Fractional ALFF. Journal of Neuroscience Methods. 2008 Jul;172(1):137–41. doi: 10.1016/j.jneumeth.2008.04.012 18501969 PMC3902859

[64] Greenhouse S. W., & Geisser S. (1959). On methods in the analysis of profile data. Psychometrika, 24(2), 95–112. 10.1007/bf02289823.

[65] Zuo X.-N., Di Martino A., Kelly C., Shehzad Z. E., Gee D. G., Klein D. F., et al. (2010). The oscillating brain: Complex and reliable. NeuroImage, 49(2), 1432–1445. doi: 10.1016/j.neuroimage.2009.09.037 19782143 PMC2856476

[66] Qi S., Gao Q., Shen J., Teng Y., Xie X., Sun Y., & Wu J. (2018). Multiple Frequency Bands Analysis of Large Scale Intrinsic Brain Networks and Its Application in Schizotypal Personality Disorder. Frontiers in Computational Neuroscience, 12. doi: 10.3389/fncom.2018.00064 30123120 PMC6085977

[67] Mason M. F., Norton M. I., Van Horn J. D., Wegner D. M., Grafton S. T., & Macrae C. N. (2007). Wandering Minds: The Default Network and Stimulus-Independent Thought. Science, 315(5810), 393–395. doi: 10.1126/science.1131295 17234951 PMC1821121

[68] Thomason M. E., Chang C. E., Glover G. H., Gabrieli J. D. E., Greicius M. D., & Gotlib I. H. (2008). Default-mode function and task-induced deactivation have overlapping brain substrates in children. NeuroImage, 41(4), 1493–1503. doi: 10.1016/j.neuroimage.2008.03.029 18482851 PMC2735193

[69] Binder J. R. (2012). Task-induced deactivation and the “resting” state. NeuroImage, 62(2), 1086–1091. doi: 10.1016/j.neuroimage.2011.09.026 21979380 PMC3389183

[70] Leech R., Kamourieh S., Beckmann C. F., & Sharp D. J. (2011). Fractionating the Default Mode Network: Distinct Contributions of the Ventral and Dorsal Posterior Cingulate Cortex to Cognitive Control. Journal of Neuroscience, 31(9), 3217–3224. doi: 10.1523/JNEUROSCI.5626-10.2011 21368033 PMC6623935

[71] McKiernan K. A., Kaufman J. N., Kucera-Thompson J., & Binder J. R. (2003). A Parametric Manipulation of Factors Affecting Task-induced Deactivation in Functional Neuroimaging. Journal of Cognitive Neuroscience, 15(3), 394–408. doi: 10.1162/089892903321593117 12729491

[72] Tommasin S., Mascali D., Gili T., Eid Assan I., Moraschi M., Fratini M., et al. (2017). Task-Related Modulations of BOLD Low-Frequency Fluctuations within the Default Mode Network. Frontiers in Physics, 5. doi: 10.3389/fphy.2017.00031 28845420 PMC5568127

[73] He Y., Wang L., Zang Y., Tian L., Zhang X., Li K., & Jiang T. (2007). Regional coherence changes in the early stages of Alzheimer’s disease: A combined structural and resting-state functional MRI study. NeuroImage, 35(2), 488–500. doi: 10.1016/j.neuroimage.2006.11.042 17254803

[74] Shen Z., Jiang L., Yang S., Ye J., Dai N., Liu X., et al. (2017). Identify changes of brain regional homogeneity in early and later adult onset patients with first-episode depression using resting-state fMRI. PLOS ONE, 12(9), e0184712. doi: 10.1371/journal.pone.0184712 28910390 PMC5598991

[75] Zuo X-N, Xu T, Jiang L, Yang Z, Cao X-Y, He Y, et al. Toward reliable characterization of functional homogeneity in the human brain: Preprocessing, scan duration, imaging resolution and computational space. NeuroImage. 2013 Jan;65:374–86. doi: 10.1016/j.neuroimage.2012.10.017 23085497 PMC3609711

[76] Zuo X-N, Xing X-X. Test-retest reliabilities of resting-state FMRI measurements in human brain functional connectomics: A systems neuroscience perspective. Neuroscience & Biobehavioral Reviews. 2014 Sep;45:100–18. doi: 10.1016/j.neubiorev.2014.05.009 24875392

[77] Di X., & Biswal B. B. (2013). Dynamic brain functional connectivity modulated by resting-state networks. Brain Structure and Function, 220(1), 37–46. doi: 10.1007/s00429-013-0634-3 25713839 PMC3980132

[78] Barnes A., Bullmore E. T., & Suckling J. (2009). Endogenous Human Brain Dynamics Recover Slowly Following Cognitive Effort. PLoS ONE, 4(8), e6626. doi: 10.1371/journal.pone.0006626 19680553 PMC2721686

[79] Akiki TJ, Averill CL, Abdallah CG. A Network-Based Neurobiological Model of PTSD: Evidence From Structural and Functional Neuroimaging Studies. Curr Psychiatry Rep. 2017 Sep 19;19(11). doi: 10.1007/s11920-017-0840-4 28924828 PMC5960989

[80] Turner BM, Rodriguez CA, Liu Q, Molloy MF, Hoogendijk M, McClure SM. On the Neural and Mechanistic Bases of Self-Control. Cerebral Cortex. 2018 Jan 24;29(2):732–50. doi: 10.1093/cercor/bhx355 29373633 PMC8921616

[81] Gagnepain P, Hulbert J, Anderson MC. Parallel Regulation of Memory and Emotion Supports the Suppression of Intrusive Memories. J Neurosci. 2017 May 30;37(27):6423–41. doi: 10.1523/JNEUROSCI.2732-16.2017 28559378 PMC5511877

[82] Wessing I, Rehbein MA, Postert C, Fürniss T, Junghöfer M. The neural basis of cognitive change: Reappraisal of emotional faces modulates neural source activity in a frontoparietal attention network. NeuroImage [Internet]. 2013 Nov;81:15–25. doi: 10.1016/j.neuroimage.2013.04.117 23664945

[83] Chen A. C., Oathes D. J., Chang C., Bradley T., Zhou Z.-W., Williams L. M., et al. (2013). Causal interactions between fronto-parietal central executive and default-mode networks in humans. Proceedings of the National Academy of Sciences, 110(49), 19944–19949. doi: 10.1073/pnas.1311772110 24248372 PMC3856839

[84] Craig A. D. (2002). How do you feel? Interoception: the sense of the physiological condition of the body. Nature Reviews Neuroscience, 3(8), 655–666. doi: 10.1038/nrn894 12154366

[85] Critchley H. D., Wiens S., Rotshtein P., Öhman A., & Dolan R. J. (2004). Neural systems supporting interoceptive awareness. Nature Neuroscience, 7(2), 189–195. doi: 10.1038/nn1176 14730305

[86] Liu C.-H., Guo J., Lu S.-L., Tang L.-R., Fan J., Wang C.-Y., et al. (2018). Increased Salience Network Activity in Patients With Insomnia Complaints in Major Depressive Disorder. Frontiers in Psychiatry, 9. doi: 10.3389/fpsyt.2018.00093 29615938 PMC5869937

[87] Andrews-Hanna J. R., Smallwood J., & Spreng R. N. (2014). The default network and self-generated thought: component processes, dynamic control, and clinical relevance. Annals of the New York Academy of Sciences, 1316(1), 29–52. doi: 10.1111/nyas.12360 24502540 PMC4039623

[88] Gerlach K. D., Spreng R. N., Gilmore A. W., & Schacter D. L. (2011). Solving future problems: Default network and executive activity associated with goal-directed mental simulations. NeuroImage, 55(4), 1816–1824. doi: 10.1016/j.neuroimage.2011.01.030 21256228 PMC3855008

[89] Meyer M. L., Spunt R. P., Berkman E. T., Taylor S. E., & Lieberman M. D. (2012). Evidence for social working memory from a parametric functional MRI study. Proceedings of the National Academy of Sciences, 109(6), 1883–1888. doi: 10.1073/pnas.1121077109 22308468 PMC3277536

[90] Beaty R. E., Benedek M., Barry Kaufman S., & Silvia P. J. (2015). Default and Executive Network Coupling Supports Creative Idea Production. Scientific Reports, 5(1). doi: 10.1038/srep10964 26084037 PMC4472024

[91] Spreng R. N., Sepulcre J., Turner G. R., Stevens W. D., & Schacter D. L. (2013). Intrinsic Architecture Underlying the Relations among the Default, Dorsal Attention, and Frontoparietal Control Networks of the Human Brain. Journal of Cognitive Neuroscience, 25(1), 74–86. doi: 10.1162/jocn_a_00281 22905821 PMC3816715

[92] Chang C., & Glover G. H. (2010). Time–frequency dynamics of resting-state brain connectivity measured with fMRI. NeuroImage, 50(1), 81–98. doi: 10.1016/j.neuroimage.2009.12.011 20006716 PMC2827259

[93] Dosenbach N. U. F., Visscher K. M., Palmer E. D., Miezin F. M., Wenger K. K., Kang H. C., et al. (2006). A Core System for the Implementation of Task Sets. Neuron, 50(5), 799–812. doi: 10.1016/j.neuron.2006.04.031 16731517 PMC3621133

[94] Petrides M. Lateral prefrontal cortex: architectonic and functional organization. Phil Trans R Soc B. 2005 Apr 29;360(1456):781–95. doi: 10.1098/rstb.2005.1631 15937012 PMC1569489

