# Supplementary material for: Dynamics of task-induced modulation of spontaneous brain activity and functional connectivity in the triple resting-state networks assessed using the visual oddball paradigm
Source: PLoS One. 2021 Nov 4;16(11):e0246709. doi: 10.1371/journal.pone.0246709 (PMC8568109; doi:10.1371/journal.pone.0246709)
Supplement: S1 Fig — Z: Cluster-corrected threshold. (DOCX) [file pone.0246709.s001.docx]

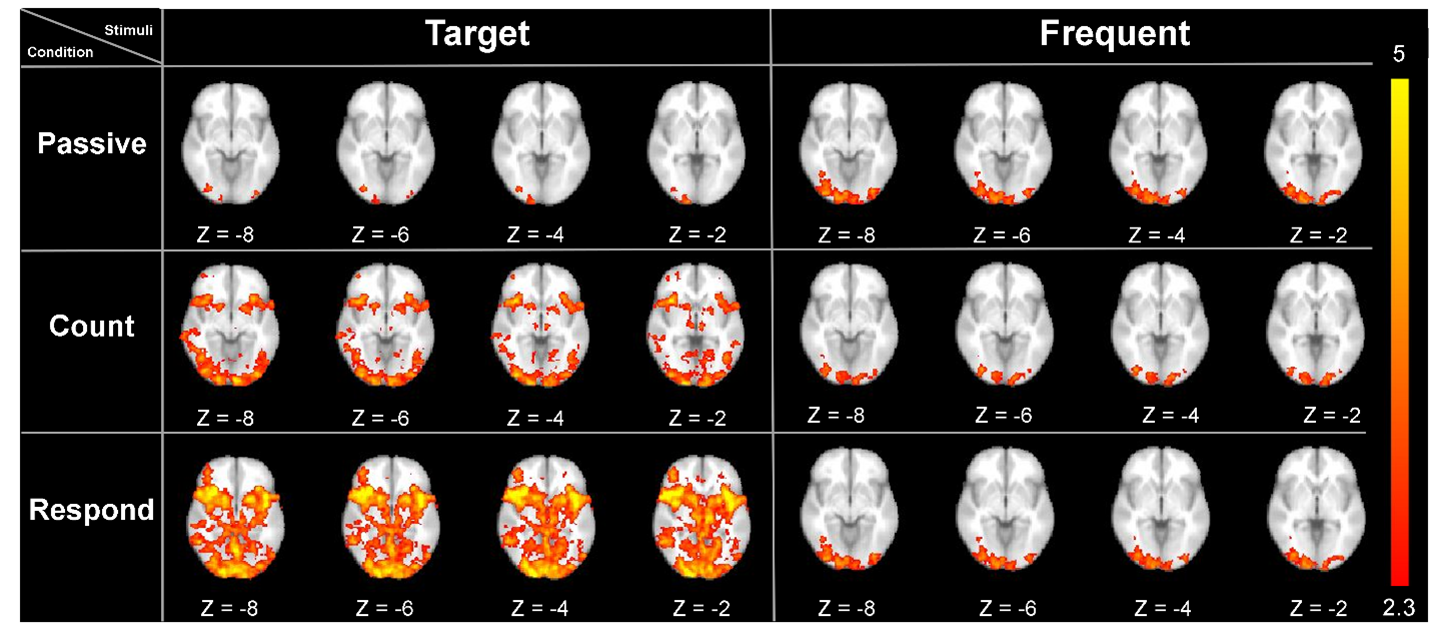


S1 Fig. BOLD activation in response to target and frequent stimuli in the passive, count and respond condition (group-level mixed-effects FLAME, single group average) for 21 healthy subjects (age: 29 ± 5.6 years), Cluster-corrected threshold (Z = 2.3, p = 0.01).
