# Supplementary material for: Dynamics of task-induced modulation of spontaneous brain activity and functional connectivity in the triple resting-state networks assessed using the visual oddball paradigm
Source: PLoS One. 2021 Nov 4;16(11):e0246709. doi: 10.1371/journal.pone.0246709 (PMC8568109; doi:10.1371/journal.pone.0246709)
Supplement: S2 Fig — Z: Cluster-corrected threshold. (DOCX) [file pone.0246709.s002.docx]

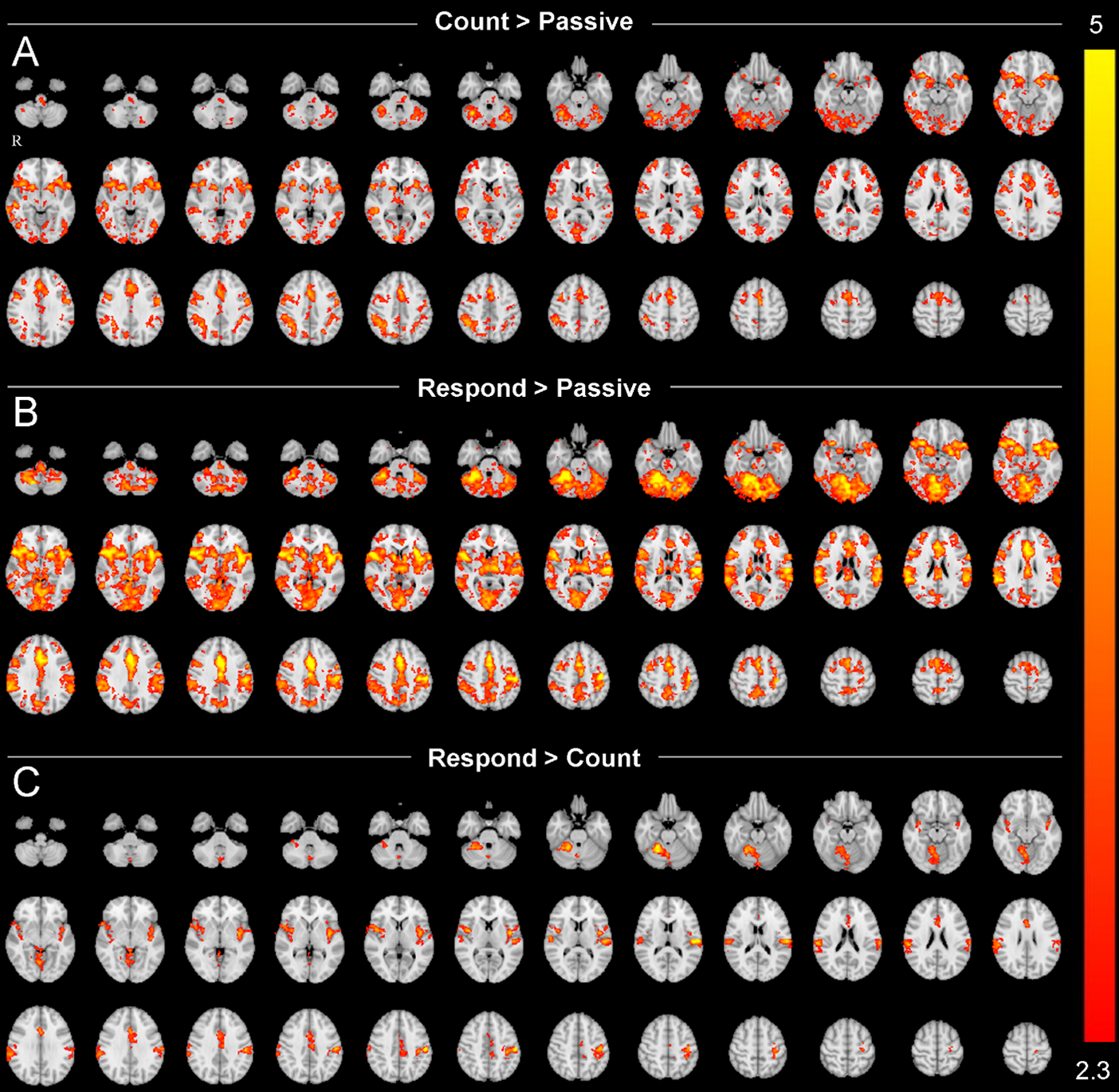


S2 Fig. BOLD activation of the tripled two-group difference (“tripled t-test”) for the (target > frequent) first level contrast. The tripled two-group differences are like follows: Count > passive (A), respond > passive (B), and respond > count (C) across 21 healthy subjects (age: 29 ± 5.6 years), Cluster-corrected threshold (Z = 2.3, p = 0.01).
